# The risk and cost of drug-drug interactions in an older population acutely admitted to hospital in Ireland

**DOI:** 10.1007/s11096-025-01907-1

**Published:** 2025-04-10

**Authors:** John E. Hughes, Kathleen E. Bennett, Caitriona Cahir

**Affiliations:** 1 School of Population Health, RCSI University of Medicine and Health Sciences, Dublin 2, Ireland; 2 Data Science Centre, School of Population Health, RCSI University of Medicine and Health Sciences, Dublin 2, Ireland

**Keywords:** Adverse drug reactions, Drug interactions, Hospital, Length of stay, Pharmacoeconomics, Pharmacoepidemiology

## Abstract

**Background:**

Adverse drug reactions (ADRs) are associated with greater healthcare costs; drug-drug interactions (DDIs) are a common cause of ADRs.

**Aim:**

To estimate the risk and cost of ADR-related hospital admission associated with DDI-exposure versus no DDI-exposure in an older community-dwelling population.

**Method:**

This is a secondary analysis of a cohort study among 798 older individuals admitted acutely to hospital in Ireland (2016–2017). Full medication-history (pre-admission) was used to measure severe DDIs. Hospital costs were derived from unit costs. Logistic regression and propensity score weighting was used to examine the association between DDI-exposure and ADR-related hospital admission. Quantile regression was used to examine median costs associated with DDI-exposure. Adjusted odds-ratios (aORs), adjusted median costs, and 95% confidence intervals (CIs) are reported.

**Results:**

N = 782 (98%) individuals using ≥ 2 drugs were included. Mean age: 80.9(SD ± 7.5) years; 52.2% female; 45.1% with an ADR-related admission. Pre-admission, n = 316 (40.4%) patients had any severe DDI, n = 466 unexposed; n = 113 (14.5%) had a DDI which increases bleeding risk, n = 669 unexposed. The risk of ADR-related hospital admission associated with any severe DDI was aOR = 1.02 [95% CI: 0.82, 1.28]), and aOR = 1.83 [95% CI: 1.35, 2.44]) for DDIs which increase bleeding risk. The median cost of ADR-related hospital admission associated with any severe DDI versus none, was €880 [− 1205, 3055]; and €3,520 [− 934, 7974] for a DDI which increases bleeding risk versus none.

**Conclusion:**

DDIs which increase bleeding risk were associated with greatest ADR-related hospital admission risk and highest costs for the healthcare system. Further research examining these DDIs is needed.

**Supplementary Information:**

The online version contains supplementary material available at 10.1007/s11096-025-01907-1.

## Impact statements

- In an older community-dwelling population, DDIs which increase the risk of bleeding were associated with a 1.8-fold greater risk and higher cost of ADR-related hospital admission.
- In clinical practice, healthcare professionals caring for the older population should be vigilant of DDIs which increase bleeding risk.
- These DDIs may potentially have broader societal and economic consequences, and further research is needed to explore this, and to identify which specific DDIs should be the focus of targeted interventions in clinical practice for the older community-dwelling population.

## Introduction

Adverse drug reactions (ADRs) are a major threat to public health and have been associated with greater healthcare costs, particularly in the older population where medication-related harm presents a growing safety and economic challenge for healthcare systems [1–3]. A 2008 European Commission pharmacovigilance report reveals that, in Europe, ADRs are the fifth most common cause of death, and their societal cost has been estimated to be approximately €79 billion [4]. More recently, a UK study reports the direct cost of ADR hospital admissions to be £490,716 [2]. Similarly, a study which used Veteran Affairs data in the United States found the median cost of severe ADRs resulting in hospitalisation to range from $6951 to $29,535 [3]. Many ADRs are, however, often caused by known drug-drug interactions (DDIs) [5, 6], such as aspirin-warfarin (causing gastrointestinal bleeding) [7, 8], concomitant use of diuretics and ACE-inhibitors (e.g., enalapril-furosemide) (precipitating renal failure) [8], and, among many others, selective serotonin re-uptake inhibitors with tricyclic antidepressants (causing serotonin syndrome) [7].

In current clinical practice, identifying and appropriately managing DDIs can be challenging; particularly in the older population, where rising medication burden, coupled with a decline in physiological reserve, independently increases an individual’s risk for potential DDI-exposure [9, 10]. Clinically important DDIs (e.g., amiodarone-warfarin) are highly prevalent in the older community-dwelling population, where estimates in European countries range from 0.8 to 54.3% [11]. Moreover, in the older (≥ 65 years) population, DDIs are reported to be responsible for between 16.6 and 49% of ADRs [7, 8, 12–14]. Previous research in the older community-dwelling population, has found potentially inappropriate prescribing (long-term non-steroidal anti-inflammatory drugs, benzodiazepines, and maximal dose proton pump inhibitors) to be significantly associated with greater healthcare costs [15]. A Canadian study in the older (≥ 65 years) community-dwelling population examined health service use and costs associated with benzodiazepine drug interactions, and found that those with one of these DDIs had a greater risk of hospitalisation and emergency and outpatient visits, and significantly higher total healthcare costs ($3,076 per year, p < 0.001) compared to older individuals with appropriate prescriptions [16]. To date, however, there is a paucity of research examining the risk and cost of ADRs associated with DDIs in the older community-dwelling population, and available studies in this area are limited to a specific patient cohort [17, 18].

### Aim

The aim of this study was to estimate the risk and cost of ADR-related hospital admission associated with DDI exposure versus no DDI exposure in an older community-dwelling population.

### Ethics approval

Ethical approval for the ADAPT study was obtained on 28/11/2016 from the Beaumont Hospital Ethics Committee (REC 16/49).

## Method

The STrengthening the Reporting of Observational Studies in Epidemiology (STROBE) and the REporting of studies Conducted using Observational Routinely collected Data for pharmacoepidemiological research (RECORD-PE) guidelines were used in the reporting of this study [19, 20].

### Study design and data source

This was a secondary analysis of a cohort study using data from the Adverse Drug Reactions in an Ageing PopulaTion (ADAPT) cohort [21]. The ADAPT study examined ADR prevalence in patients aged ≥ 65 years admitted acutely from the community to a large (820-bed) tertiary referral hospital in Dublin, Ireland, between November 2016 and June 2017, and prospectively recorded patient-reported health outcomes and costs associated with these hospital admissions [21]. Data on ADR- and non-ADR-related hospital admissions in 798 individuals aged ≥ 65 years were analysed. For all study participants, baseline data on sociodemographics, functional ability, and chronic disease (from medical records, coded using the ICD-10 classification) were available. Full medication data (current, recently discontinued, over-the-counter) preceding hospital admission, and coded at the fifth level of the World Health Organization (WHO) Anatomical Therapeutic Chemical (ATC) classification system, as well as objective clinical data (e.g., creatinine), were available. Linked pharmacy claims data in the 12-months pre- and post-hospital index admission date were available for General Medical Services (GMS) eligible study participants who provided consent (n = 255). The ADAPT study is described in detail elsewhere [21, 22].

### Drug-drug interaction exposure

Based on findings from our previous study [23], the exposure of interest was (i) any severe (i.e. the result may be a life-threatening event or have a permanent detrimental effect) potentially clinically important DDI versus none; and (ii) DDIs which increase the risk of bleeding versus none. These DDIs were identified from a list of 28,225 severe potentially clinically important DDIs extracted from two comprehensive drug interaction references: the British National Formulary 84 and Stockley's Drug Interactions [23]. Medication data for all ADAPT study participants using two or more medications (N = 782) were analysed. Unless otherwise stated, any reference made herein to a DDI refers to those classified as "severe" potentially clinically important.

### ADR-related hospital admissions

All acutely admitted patients in ADAPT were screened, within 36 h of admission, for a suspected ADR-related hospital admission by the clinical research team (composed of a consultant geriatrician, two hospital pharmacists, and a research nurse) using a previously validated screening process [8, 12]. ADR causality was determined using the World Health Organisation (WHO) criteria, the Naranjo criteria, and the Liverpool Algorithm [22]. A random sample of patients, admitted to hospital without a suspected ADR, were assigned to a non-ADR control group for comparison.

### Cost of ADR-related hospital admission

The cost for in-hospital services was estimated for each ADAPT patient. We used unit costs reported by Beaumont Hospital (on average, approximately €880 per bed-day). The overall hospital admission cost per patient was expressed per total length of stay (LOS) in hospital, defined as the duration of time (in days) spent in hospital from date of admission to discharge.

### Covariates

The following potential covariates were available: age (continuous); gender (male/female); smoker (yes/no); medical card (yes/no); frailty (continuous; measured using the PRISMA-7); fall in the past year (yes/no); multiple falls in the past year (yes/no); delirium (yes/no; measured using the DSM-IV); co-morbidities (yes/no); Charlson Comorbidity Index ([1] 0; [2] 1–2 [3]; ≥ 3); renal impairment (estimated using creatinine clearance and the Cockcroft-Gault equation; classified as: [1] mild; [2] moderate; [3] severe; [4] end-stage renal disease [ESRD]); medications coded at Anatomical Therapeutic Chemical (ATC) level 3 (yes/no); polypharmacy ([1] no; [2] polypharmacy; [3] major polypharmacy); narrow therapeutic index drug (yes/no).

### Statistical analysis

Baseline characteristics of the population are reported as means (± SD) for continuous variables and as number of patients (%) for categorical variables. DDI prevalence estimates and 95% confidence intervals (CIs) are presented. To minimise selection bias, propensity scores (for DDI exposure) were estimated using logistic regression. The propensity scores were used to calculate stabilised (truncated at the 1st and 99th percentile) [24] inverse probability of treatment weights (IPTW), to re-weight both DDI and non-DDI groups to the propensity score distribution of the full population. Standardised mean differences (SMD) were used to assess covariate balance [25]. The association between DDI exposure and ADR-related hospital admission was estimated using a logistic regression model with stabilised IPTW. Previously constructed directed acyclic graphs [23] were used to identify confounders and prognostic variables to include in outcome regression models. Duration of potential DDI exposure was investigated by analysing pharmacy claims data for consenting ADAPT participants using two or more medications (n = 254). The association between DDI exposure and ADR-related hospital admission (median) costs was estimated using quantile regression, due to skewness/heteroskedasticity in the dependent variable [26], with stabilised IPTW. Sensitivity analyses were conducted for robustness, using 1:1 nearest neighbour propensity score matching, with a 0.1 caliper and without replacement, to examine the risk and cost of DDI exposure associated with ADR-related hospital admissions. Adjusted odds-ratios (aORs) with 95% bootstrapped (1000 samples) CIs, estimated using robust standard errors (RSE), are reported for the risk of ADR-related hospital admission. Adjusted median costs with 95% CIs (exponentiated from regression) are also reported. Analysis was performed using SAS version 9.4 statistical package and R statistical software (version 4.1.2).

## Results

### Study population

In total, 798 participants were included in the ADAPT study; 782 (98.0%) used two or more drugs and were included for analysis. Pre-hospital admission, n = 316 (40.4% [95% CI: 37.0, 43.9]) patients were potentially exposed to at least one (range: 1–15) potentially clinically important DDI, n = 466 patients had no DDI. Prevalent DDIs included: aspirin-warfarin (n = 37); bisoprolol-lidocaine (n = 33); amiodarone-furosemide (n = 21); amiodarone-bisoprolol (n = 18); aspirin-apixaban (n = 17). After IPTW, baseline covariates were well-balanced (SMD < 0.1) [27] (Table 1); and there was good overlap in the propensity score distribution for DDI and non-DDI groups (Figure S1). Pre-hospital admission, n = 113 (14.5% [12.2, 17.1]) patients were potentially exposed to at least one (range: 1–3) DDI which increases the risk of bleeding; n = 669 were unexposed. Prevalent DDIs which increase the risk of bleeding included: aspirin-warfarin (n = 37); aspirin-apixaban (n = 17); amiodarone-warfarin (n = 13); rosuvastatin-warfarin (n = 9); and aspirin-nicorandil (n = 7). After IPTW, baseline covariates were well-balanced (SMD < 0.1) [27] (Table 2), and there was good overlap in the propensity score distribution for DDI and non-DDI groups (Figure S2). Among the n = 254 patients with pharmacy claims data, DDI prevalence in the 12-month period pre-hospital admission date was comparable to that observed on admission to hospital (Table S1). In addition, the most prevalent DDIs identified on admission to hospital were found to be consistently dispensed across patients, during the 12-month period pre-hospital admission date (Figure S3).

**Table 1:** Any severe drug-drug interaction: Patient characteristics before and after inverse probability of treatment weighting (IPTW)

	Any Severe DDI					
	Before IPTW			After IPTW		
	DDI (n = 316)	No DDI (n = 466)	SMD*	DDI (n = 268)^#^	No DDI (n = 355)^#^	SMD*
*Sociodemographics*						
Age (mean [± SD])	81.2 (7.3)	80.7 (7.7)	0.07	80.0 (7.8)	80.7 (7.6)	− 0.09
Female	0.54	0.51	0.85	0.49	0.52	− 0.03
Smoker	0.58	0.59	− 0.01	0.59	0.59	0.00
Medical Card	0.39	0.33	0.12	0.38	0.36	0.02
*Functional ability*						
Frailty (PRISMA-7)	0.66	0.55	0.11	0.57	0.59	− 0.02
Fall in past year	0.65	0.73	0.08	0.67	0.69	− 0.01
Multiple falls in past year	0.83	0.88	0.05	0.87	0.86	0.01
Delirium (DSM-IV)	0.35	0.29		0.33	0.30	0.03
*ICD-10 Co-Morbidities*						
Cancer	n < 5	~		0.01	0.01	0.00
Cerebrovascular disease	0.11	0.11	0.00	0.12	0.13	0.00
Chronic kidney disease	0.06	0.03	0.07	0.05	0.04	0.01
Chronic liver disease	0.00	0.00	0.00	0.00	0.00	0.00
Chronic lung disease	0.14	0.14	0.00	0.12	0.13	− 0.01
Connective tissue disease	0.04	0.04	0.00	0.03	0.04	− 0.01
Dementia	0.07	0.09	− 0.03	0.07	0.08	− 0.01
Diabetes mellitus	0.03	0.02	0.02	0.02	0.02	0.00
Heart failure	0.18	0.08	0.28	0.13	0.10	0.03
Myocardial infarction	0.15	0.09	0.12	0.11	0.11	0.00
Ulcer disease	0.02	0.03	− 0.02	0.02	0.03	0.00
Charlson Comorbidity Index			0.42			0.09
0	0.09	0.21		0.16	0.17	
1–2	0.39	0.45		0.39	0.43	
≥ 3	0.52	0.34		0.45	0.40	
Renal impairment (CrCl)			0.25			− 0.01
Mild (50–80 mL/min)	0.31	0.41		0.33	0.40	
Moderate (30–49 mL/min)	0.35	0.27		0.34	0.30	
Severe (15–29 mL/min)	0.18	0.11		0.13	0.12	
ESRD (< 15 mL/min)	0.04	0.05		0.03	0.05	
*Medication (ATC code)*						
Anticoagulant (B01A)	0.48	0.17	0.75	0.31	0.24	0.07
Antiplatelet (B01AC)	0.63	0.53	0.23	0.55	0.57	− 0.02
Diuretic (C03)	0.58	0.33	0.53	0.45	0.39	0.07
Antiarrhythmics, Class I and III (C01B)	~	n < 5		0.06	0.02	0.04
Beta blocking agents (C07A)	0.64	0.41	0.55	0.50	0.47	0.03
Calcium channel blockers (C08)	0.29	0.27	0.05	0.24	0.27	− 0.03
RAAS (C09)	0.50	0.47	0.07	0.45	0.48	− 0.03
Lipid modifying agents, plain (C10A)	0.77	0.63	0.28	0.70	0.67	0.03
NSAID (M01A)	0.05	0.08	− 0.10	0.06	0.07	− 0.01
Antidepressants (N06A)	0.38	0.20	0.34	0.33	0.27	0.06
Anti-dementia drugs (N06D)	0.10	0.09	0.02	0.09	0.09	0.00
Hypnotics and sedatives (N05C)	0.22	0.18	0.08	0.21	0.19	0.02
Analgesics (N02)	0.46	0.35	0.22	0.40	0.39	0.01
Polypharmacy			0.95			0.22
No (0–4 drugs)	n < 5	~		0.03	0.08	
Polypharmacy (5–9 drugs)	0.18	0.48		0.45	0.38	
Major polypharmacy (≥ 10 drugs)	0.81	0.39		0.61	0.54	
Narrow Therapeutic Index drug^†^	0.64	0.44	0.19	0.55	0.50	0.05

Data presented as mean values/proportions, unless otherwise stated

^*^SMD < 0.1 indicates balance between groups

^#^Reflects sample size after trimming and weighting

~ Data further suppressed to prevent disclosure of data where n < 5

^†^Carbamazepine, digoxin, flecainide, lithium, phenytoin, tacrolimus, theophylline, warfarin

Abbreviations: ATC, Anatomical Therapeutic Chemical; CrCl, creatinine clearance; ESRD, end-stage renal disease; SMD, standardised mean difference

**Table 2:** Drug-drug interaction which increases bleeding risk: Patient characteristics before and after inverse probability of treatment weighting (IPTW)

	DDI which Increases Bleeding Risk					
	Before IPTW			After IPTW		
	DDI (n = 113)	No DDI (n = 669)	SMD*	DDI (n = 102)^#^	No DDI (n = 535)^#^	SMD*
*Sociodemographics*						
Age (mean [± SD])	81.13 (7.13)	80.83 (7.62)	0.04	80.80 (7.69)	80.87 (7.72)	− 0.08
Female	0.49	0.53	− 0.04	0.49	0.52	− 0.03
Smoker	0.62	0.58	0.04	0.55	0.58	− 0.03
Medical Card	0.41	0.35	0.06	0.55	0.57	− 0.02
*Functional ability*						
Frailty (PRISMA-7)	0.69	0.58	0.11	0.53	0.58	− 0.05
Fall in past year	0.64	0.70	− 0.07	0.54	0.54	0.00
Multiple falls in past year	0.86	0.86	0.00	0.83	0.87	− 0.03
Delirium (DSM-IV)	0.34	0.31	0.03	0.22	0.30	− 0.09
*ICD-10 Co-Morbidities*						
Cancer	0.00	0.01	− 0.01	0.00	0.01	− 0.01
Cerebrovascular disease	0.12	0.11	0.02	0.22	0.20	0.02
Chronic kidney disease	n < 5	~	− 0.03	0.01	0.05	− 0.04
Chronic liver disease	0.00	0.00	0.00	0.00	0.00	0.00
Chronic lung disease	0.12	0.14	− 0.02	0.16	0.14	0.03
Connective tissue disease	n < 5	~	0.00	0.02	0.04	− 0.02
Dementia	0.04	0.08	− 0.04	0.02	0.08	− 0.06
Diabetes mellitus	0.04	0.02	0.01	0.02	0.02	0.00
Heart failure	0.21	0.10	0.11	0.10	0.10	0.00
Myocardial infarction	0.25	0.09	0.16	0.27	0.22	0.05
Ulcer disease	n < 5	~	− 0.01	0.01	0.03	− 0.02
Charlson Comorbidity Index			0.33			0.01
0	0.10	0.17		0.13	0.16	
1–2	0.35	0.44		0.51	0.45	
≥ 3	0.55	0.39		0.36	0.39	
Renal impairment (CrCl)			0.34			− 0.08
Mild (50–80 mL/min)	0.28	0.39		0.21	0.14	
Moderate (30–49 mL/min)	0.35	0.30		0.31	0.38	
Severe (15–29 mL/min)	0.27	0.12		0.33	0.31	
ESRD (< 15 mL/min)	n < 5	~		0.14	0.13	
*Medication (ATC code)*						
Anticoagulant (B01A)	0.93	0.19	0.74	0.51	0.47	0.04
Antiplatelet (B01AC)	0.73	0.55	0.18	0.74	0.66	0.07
Diuretic (C03)	0.58	0.41	0.18	0.68	0.58	0.09
Antiarrhythmics, Class I and III (C01B)	0.19	0.03	0.16	0.09	0.06	0.03
Beta blocking agents (C07A)	0.75	0.46	0.29	0.71	0.62	0.08
Calcium channel blockers (C08)	0.28	0.28	0.01	0.37	0.28	0.09
RAAS (C09)	0.55	0.47	0.08	0.66	0.59	0.07
Lipid modifying agents, plain (C10A)	0.75	0.67	0.08	0.84	0.79	0.05
NSAID (M01A)	0.06	0.07	0.00	0.04	0.06	− 0.02
Antidepressants (N06A)	0.30	0.27	0.03	0.26	0.26	0.00
Anti-dementia drugs (N06D)	0.07	0.10	-0.03	0.04	0.09	− 0.05
Hypnotics and sedatives (N05C)	0.19	0.20	0.00	0.20	0.20	0.00
Analgesics (N02)	0.35	0.41	− 0.05	0.33	0.40	− 0.07
Polypharmacy			0.56			0.09
No (0–4 drugs)	n < 5	~		0.04	0.08	
Polypharmacy (5–9 drugs)	0.23	0.38		0.31	0.36	
Major polypharmacy (≥ 10 drugs)	0.76	0.53		0.75	0.66	
Narrow Therapeutic Index drug^†^	0.79	0.48	0.31	0.66	0.61	0.05

Data presented as mean values/proportions, unless otherwise stated

^*^SMD < 0.1 indicates balance between groups

^#^Reflects sample size after trimming and weighting

~ Data further suppressed to prevent disclosure of data where n < 5

^†^Carbamazepine, digoxin, flecainide, lithium, phenytoin, tacrolimus, theophylline, warfarin

Abbreviations: ATC, Anatomical Therapeutic Chemical; CrCl, creatinine clearance; ESRD, end-stage renal disease; SMD, standardised mean difference

### Risk of ADR-related hospital admission

Overall, n = 353 (45.1%) patients had an ADR-related admission. Among those with any severe potentially clinically important DDI pre-admission, n = 156 (49.4%) had an ADR-related admission; among those with a pre-admission DDI which increases the risk of bleeding, n = 70 (62.0%) had an ADR-related admission. The risk of ADR-related hospital admission associated with any severe DDI exposure was estimated to be aOR = 1.02 [95% CI: 0.82, 1.28]). For patients exposed to a DDI which increases the risk of bleeding, the risk was estimated to be aOR = 1.83 [95% CI: 1.35, 2.44]). These findings were robust to sensitivity analysis, see supplementary material. Among patients with pharmacy claims data, n = 103 (40.6%) had an ADR-related hospital admission. Among those with any severe DDI, n = 62 (42.8%) had an ADR-related admission; among those with a DDI which increases the risk of bleeding, n = 24 (20.7%) had an ADR-related admission.

### Cost of ADR-related hospital admission

In the overall population (N = 782), the median LOS in hospital was 7 [IQR 4–15] days, and the median cost of hospital admission was €6,160 [IQR 3520–13,200]. Any severe DDI exposure was associated with a median cost of €880 [− 1,205, 3,055] for ADR-related hospital admission, compared to no DDI exposure. The median cost of ADR-related hospital admission associated with exposure to a DDI which increases the risk of bleeding, compared to those without one of these DDIs, was €3,520 [− 934, 7,974] (Table 3). These findings were robust to sensitivity analysis, see supplementary material.

**Table 3:** Cost (in Euro) of ADR-Related Hospital Admission by Type of DDI

	Median Cost of ADR-Related Hospital Admission (€)	95% CI (€)
Any Severe† DDI*	880	[− 1250, 3055]
DDI which Increases Bleeding Risk**	3520	[− 934, 7974]

^*†*^The result may be a life-threatening event or have a permanent detrimental effect

^*^GBP: £756 [− 1074, 2624]; USD: $949 [− 1348, 3295]

^**^GBP: £3024 [− 802, 6850]; USD: $3797 [− 1008, 8602]

Based on average exchange rate between Nov2016 and Jun2017: (1EUR: 0.8590GBP); (1EUR: 1.0787USD)

## Discussion

### Key findings

In a population of older adults admitted acutely from the community to a large hospital in Ireland, this study found that DDIs which increase bleeding risk were associated with greatest risk of ADR-related hospital admission and highest cost for the healthcare system. These findings were robust to sensitivity analyses.

### Strengths and weaknesses

This study used real-world data from the ADAPT cohort, which includes data that are not routinely available in administrative claims databases. This included hospital unit costs and duration of time spent in hospital; as well as objective clinical data to measure renal function (i.e., creatinine clearance), using the Cockcroft-Gault equation, which is most appropriate in this older population [28]. The medication data used in this study included current, recently discontinued, and over-the-counter medication, reconciled from multiple sources. This study also benefited from access to national pharmacy claims data, though for a subset of the population. Compared to other studies, we also examined a large number of potentially clinically important DDIs (N = 28,225). However, our study has limitations which should be considered. Firstly, medication adherence has been associated with ADRs [29], which may have influenced our findings. In the ADAPT cohort, 93.3% of patients in the ADR-related hospital admission group had self-reported adherence [22]. However, adherence based on pharmacy claims data may reveal different patterns, and this should be considered when interpreting our findings. We analysed pharmacy claims data for consenting ADAPT participants to examine the duration of potential DDI exposure pre-hospital admission, and found that prevalence estimates, overall and for individual-level DDIs, were broadly comparable to that observed on admission. However, we were unable to distinguish between pre-existing and admission-related DDIs for all patients in the study population, and our results should be interpreted with this in mind. Due to study power limitations, it was not possible to examine the risk and cost associated with individual level DDIs (e.g., aspirin-apixaban); and larger studies are therefore needed to investigate specific high-risk DDIs. In addition, the data used in this study were collected in 2016–2017, and longitudinal follow-up and monitoring of DDIs at the population-level is needed for more conclusive evidence in this older population. The data used in this study were limited to older individuals in Ireland, and the results may not be generalisable to other developed countries. Our study assumes that all reported or dispensed medications were taken.

### Interpretation

The findings from this study suggest that, in an older community-dwelling population, while many of the severe DDIs examined did not appear to increase the overall risk of ADR-related hospital admission, DDIs which increase bleeding risk were associated with greatest risk and cost of ADR-related hospital admission. These findings align with previous research which has found DDIs involving anticoagulants (e.g., aspirin-warfarin causing gastrointestinal bleeding) to be implicated in ADRs requiring hospital admission [7, 8, 30]. Previous studies which have evaluated the cost of adverse health outcomes associated with DDIs in the older community-dwelling population, have also reported increased cost associated with DDIs [17, 18]; however, due to differences in study population and the type of DDIs examined, direct comparison of findings is challenging. A cross-sectional study in Germany among older (≥ 70 years) community-dwelling individuals with dementia examined the association between drug-related problems (including potential clinically relevant and moderately severe DDIs), and found a non-significant increase in the cost for hospitalisation (€88 [95% CI: − 1023 to 1200]) [18]. A recent UK study reports an extrapolated projected annual direct cost of ADR admissions to the National Health Service of £2.21 billion, with 29.4% of ADRs potentially caused by a DDI; though details of the specific DDIs involved were not provided [2]. Our findings contribute to the current literature on high-risk medication in the older community-dwelling population by highlighting the higher costs for ADR-related hospital admission associated with severe DDIs, in particular DDIs which increase the risk of bleeding. In clinical practice, greater vigilance among healthcare professionals caring for this older population is needed to mitigate exposure to DDIs which increase the risk of bleeding. However, further research is needed to validate these findings and to identify which specific DDIs should be the focus of targeted interventions in the older community-dwelling population.

### Further research

Our findings provide insight into specific DDIs which should be investigated in future health-economics outcomes research to assess their potential risk and cost in the older community-dwelling population. This requires large data, with longitudinal follow-up, and well-designed studies to mitigate multiple sources of bias inherent to DDI pharmacoepidemiology research [31]. Specifically, future health-economic outcomes research should investigate the prevalent DDIs identified by our study, as well as DDIs which increase the risk of bleeding, using the target trial emulation framework to ensure robust study design, and to yield meaningful effect estimates [32]. This could also be achieved through routine pharmacovigilance monitoring at the population-level, using large longitudinal data and objective clinical endpoints. This would facilitate the proactive identification and characterisation of potentially avoidable medication-related harm involving DDIs in the older population. There is also a need for cross-country collaborative research to evaluate health outcomes and costs associated with DDIs in the older community-dwelling population for comparison across different health systems. Finally, further research using a longitudinal cohort study design is needed to understand the economic burden of DDIs from the societal perspective.

## Conclusion

DDIs which increase the risk of bleeding were associated with an approximate two-fold increased risk of ADR-related hospital admission, and represent substantial additional cost for the healthcare system. Future health-economics outcomes research should focus on these specific DDIs in this older population.

## Supplementary Information

Below is the link to the electronic supplementary material.

Supplementary file1 (DOCX 3035 KB)
