# Supplementary material for: The risk and cost of drug-drug interactions in an older population acutely admitted to hospital in Ireland
Source: Int J Clin Pharm. 2025 Apr 10;47(5):1239–47. doi: 10.1007/s11096-025-01907-1 (PMC12432064; doi:10.1007/s11096-025-01907-1)
Supplement: Supplementary file 1 — Supplementary file1 (DOCX 3035 KB) [file 11096_2025_1907_MOESM1_ESM.docx]

**Electronic Supplementary Material**

**Article title:** The Risk and Cost of Drug-Drug Interactions in an Older Population acutely admitted to Hospital in Ireland.

**Authors:** John E. Hughes^1^, Kathleen E. Bennett^2^, Caitriona Cahir^2^

**Journal name:** International Journal of Clinical Pharmacy

**Affiliations & Institutions:**

^1^School of Population Health, RCSI University of Medicine and Health Sciences, Dublin 2, Ireland.

^2^Data Science Centre, School of Population Health, RCSI University of Medicine and Health Sciences, Dublin 2, Ireland.

**Corresponding Author:**

John E. Hughes;

School of Population Health, Royal College of Surgeons in Ireland, Dublin 2;

Email: [johnehughes@rcsi.com](mailto:johnehughes@rcsi.com) ; [hughesjo@tcd.ie](mailto:hughesjo@tcd.ie)

ORCID: <https://orcid.org/0000-0002-3944-8326>

Table of Contents

[Propensity Scores for Inverse Probability of Treatment Weights 3](#_Toc190276410)

[**Figure S1.** Propensity Score Distribution Before and After Weighting, for Any Severe DDI and non-DDI groups. 3](#_Toc190276411)

[**Figure S2.** Propensity Score Distribution Before and After Weighting, for DDI which Increases Bleeding Risk and non- DDI which Increases Bleeding Risk groups. 3](#_Toc190276412)

[Duration of potential DDI exposure 4](#_Toc190276413)

[DDI Prevalence Pre-Hospital Admission 4](#_Toc190276414)

[**Table S1.** DDI prevalence at Hospital Admission and 12-Months Pre-Hospital Admission for ADAPT patients with linked pharmacy claims data 4](#_Toc190276415)

[**Figure S3.** Top 10 DDIs identified at hospital admission and their prevalence in the 12-month pre-hospital admission period based on linked pharmacy claims data. 4](#_Toc190276416)

[Sensitivity Analyses Using Propensity Score Matching 5](#_Toc190276417)

[Any Severe Potentially Clinically Important DDI 5](#_Toc190276418)

[Study Population 5](#_Toc190276419)

[6](#_Toc190276420)

[**Figure S4.** Distribution of the estimated propensity score for DDI (any) exposure, before and after matching 6](#_Toc190276421)

[**Table S2.** Patient Characteristics Before and After Propensity Score Matching (Any Severe Potentially Clinically Important DDI) 7](#_Toc190276422)

[DDIs which Increase Bleeding Risk 9](#_Toc190276423)

[Study Population 9](#_Toc190276424)

[10](#_Toc190276425)

[**Figure S5.** Distribution of the estimated propensity score for DDI (with increased bleeding risk) exposure, before and after matching 10](#_Toc190276426)

[**Table S3.** Patient Characteristics Before and After Propensity Score Matching (DDI which Increases Bleeding Risk) 11](#_Toc190276427)

[Risk of ADR-Related Hospital Admission 13](#_Toc190276428)

[Cost of ADR-Related Hospital Admission 14](#_Toc190276429)

[**Table S5.** Sensitivity analysis using propensity score matching, cost (in Euro) of ADR-Related Hospital Admission by Type of DDI 14](#_Toc190276430)

# Propensity Scores for Inverse Probability of Treatment Weights


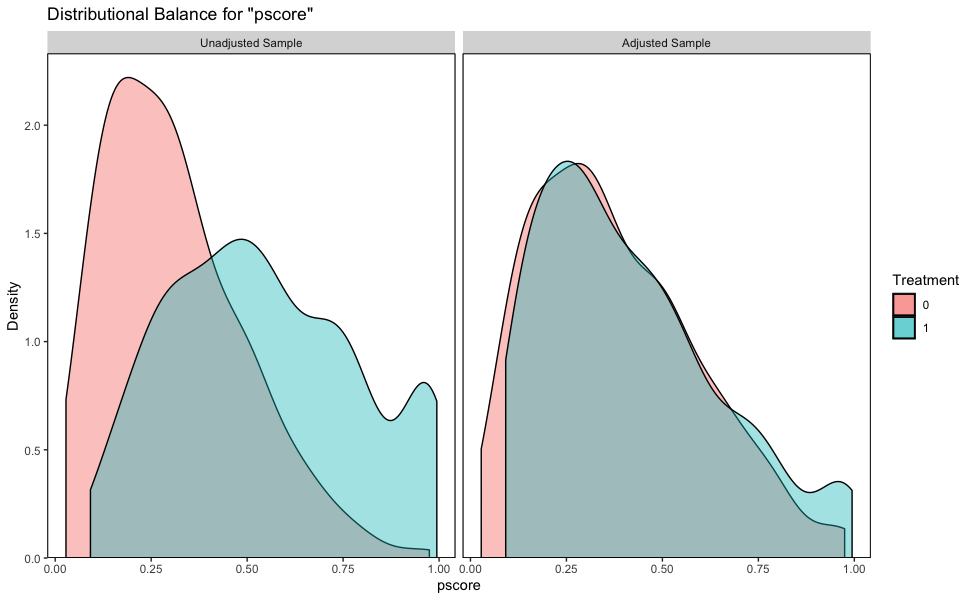


### **Figure S1.** Propensity Score Distribution Before and After Weighting, for Any Severe DDI and non-DDI groups.


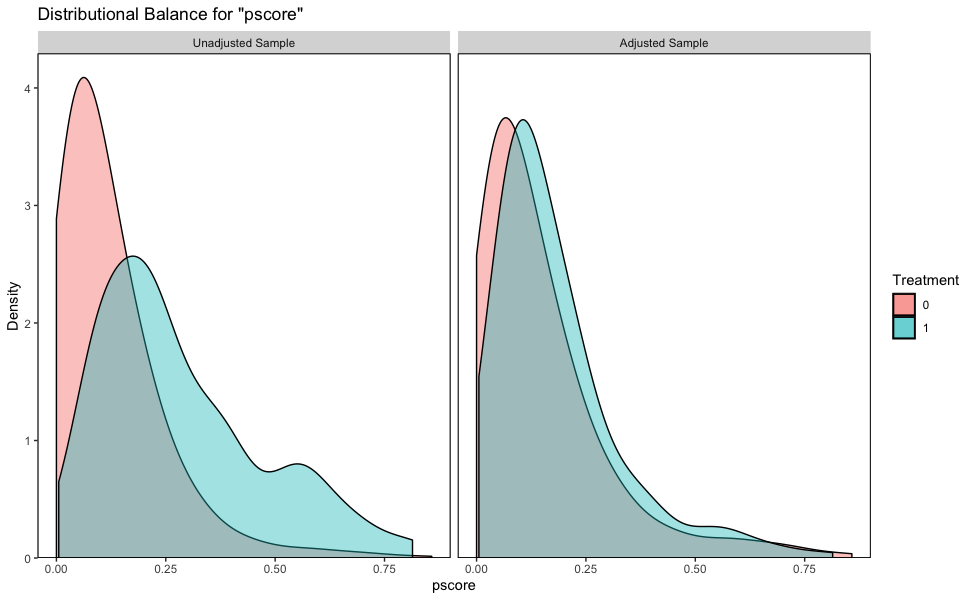


### **Figure S2.** Propensity Score Distribution Before and After Weighting, for DDI which Increases Bleeding Risk and non- DDI which Increases Bleeding Risk groups.

# Duration of potential DDI exposure

## DDI Prevalence Pre-Hospital Admission

Pharmacy claims data were available for n=254 ADAPT patients. In the 12-month period prior to hospital admission, n=145 (57.1% [95%CI: 50.9, 63.0]) patients were continuously dispensed at least one severe potentially clinically important DDI; n=116 (45.7%) were continuously dispensed at least one DDI which increases bleeding risk (***Table S1***). Among patients with any severe DDI, n=62 (42.8%) had an ADR-related hospital admission; among those with a DDI which increases the risk of bleeding, n=24 (20.7%) had an ADR-related admission. ***Figure S3*** provides a summary of the top 10 most prevalent DDIs on admission to hospital and in the 12-month pre-hospital admission period, for ADAPT with pharmacy claims data.

### **Table S1.** DDI prevalence at Hospital Admission and 12-Months Pre-Hospital Admission for ADAPT patients with linked pharmacy claims data

|  | **12-Months Pre-Hospital Admission (n=254)** | | **On Admission to Hospital (n=254)** | |
| --- | --- | --- | --- | --- |
|  | **n (%)** | **[95% CI]** | **n (%)** | **[95% CI]** |
| Any Severe† DDI | 145 (57.1) | [50.9, 63.0] | 116 (45.7) | [39.7, 51.8] |
| DDI which Increases Bleeding Risk | 47 (18.5) | [14.2, 23.8] | 44 (17.3) | [13.1, 22.5] |
| *†The result may be a life-threatening event or have a permanent detrimental effect.*  *Abbreviations: DDI, Drug-Drug Interaction.* | | | | |

**
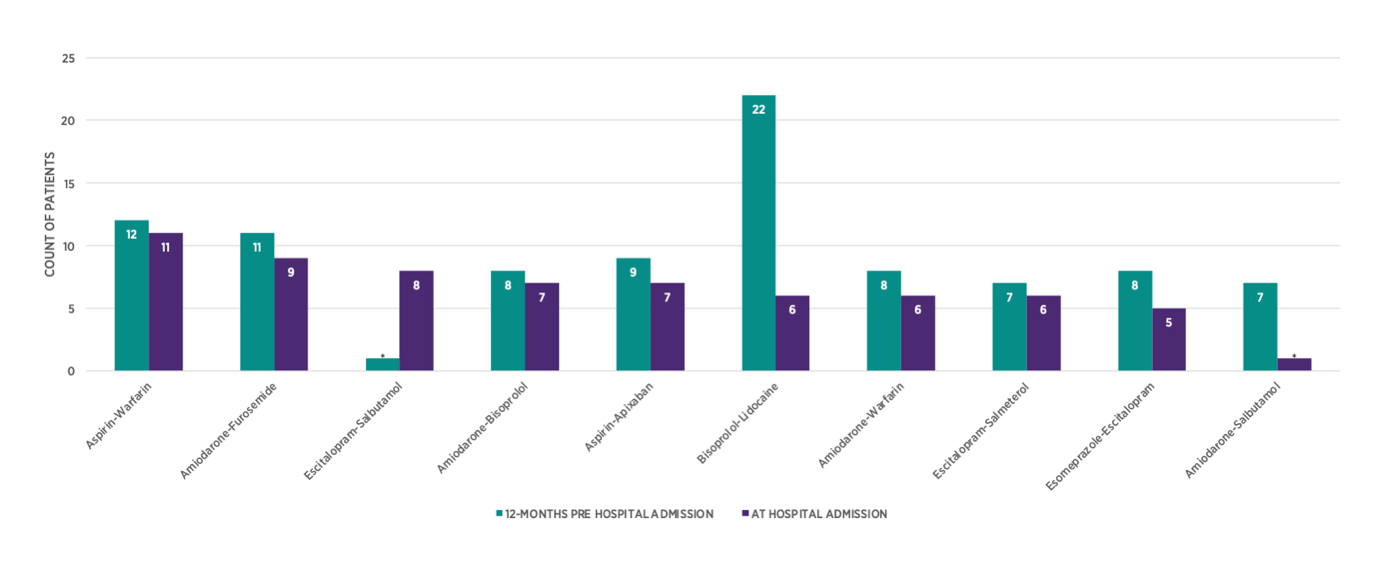
**

### **Figure S3.** Top 10 DDIs identified at hospital admission and their prevalence in the 12-month pre-hospital admission period based on linked pharmacy claims data.

*data where n<5 not presented.

# Sensitivity Analyses Using Propensity Score Matching

Two separate propensity score matching models were constructed for: (1) any severe potentially clinically important DDI; and (2) DDIs which increase an individual’s risk of bleeding. For any severe potentially clinically important DDI, from the 782 ADAPT patients eligible for inclusion, 462 (n=231 DDI and n=231 non-DDI) were successfully matched. For DDIs which increase the risk of bleeding, from the 782 ADAPT patients eligible for inclusion, 226 (n=113 DDI and n=113 non-DDI) were successfully matched. Full details on both propensity score matched populations are described below.

## Any Severe Potentially Clinically Important DDI

### Study Population

For any potentially clinically important DDI, from the 782 ADAPT patients eligible for inclusion, 462 (n=231 DDI and n=231 non-DDI) were successfully matched. After propensity score matching, the DDI-exposed and non-DDI exposed groups had similar propensity score distributions, with good overlap was achieved (**figure S3**); and confounders in the causal relationship between DDI exposure and ADR-related hospital admission were well-balanced (**Table S1**). The mean age of the matched sample was 81 (±7.5) years; 52% were female; 32.5% had mild, 36.4% had moderate, and 13.0% had severe renal impairment; 25.1% had polypharmacy, 73.6% had major polypharmacy; and 55.0% had at least one NTI drug (**Table S1**).

###


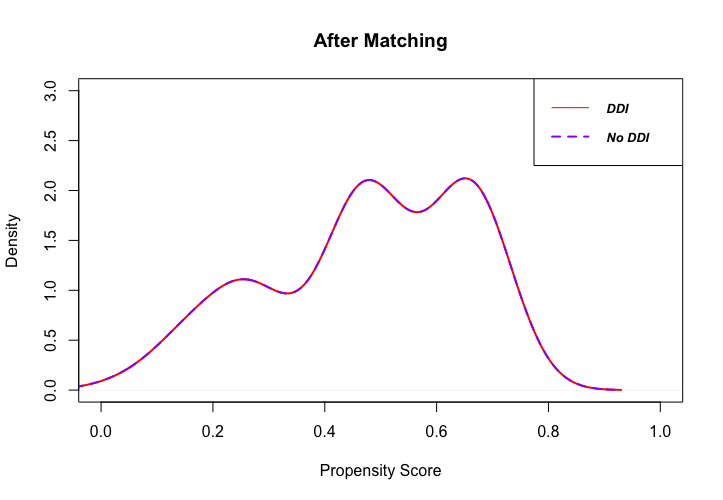

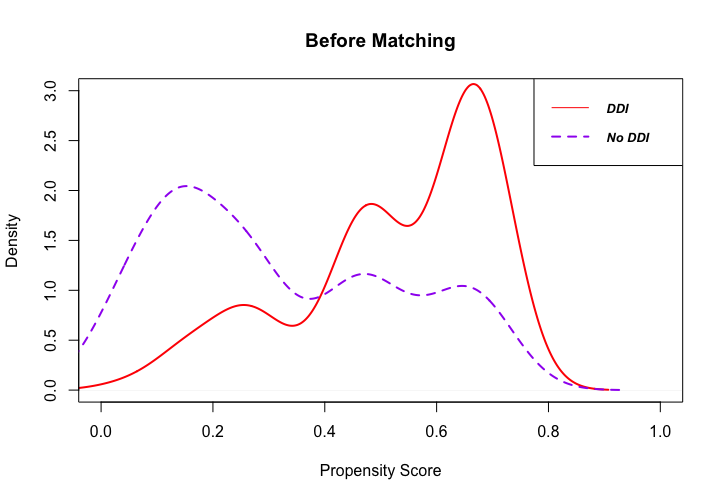


### **Figure S4.** Distribution of the estimated propensity score for DDI (any) exposure, before and after matching

### **Table S2.** Patient Characteristics Before and After Propensity Score Matching (Any Severe Potentially Clinically Important DDI)

|  | **Before Matching** | | | | **After Matching** | | |
| --- | --- | --- | --- | --- | --- | --- | --- |
|  | **Overall (N=782)** | **DDI (n=316)** | **No DDI (n=466)** | **SMD** | **DDI (n=231)** | **No DDI (n=231)** | **SMD** |
| **Sociodemographics** |  |  |  |  |  |  |  |
| Age (mean [SD]) | 80.9 (±7.5) | 81.2 (±7.3) | 80.7 (±7.7) | 0.07 | 80.5 (±7.2) | 80.8 (±7.7) | 0.04 |
| *Female* | 408 (52.2) | 170 (53.8) | 238 (51.1) | 0.06 | 120 (51.9) | 120 (51.9) | <0.001 |
| Smoker | 456 (58.3) | 183 (57.9) | 273 (58.6) | 0.01 | 124 (53.7) | 129 (55.8) | 0.07 |
| Medical Card | 277 (35.4) | 124 (39.2) | 153 (32.8) | 0.13 | 86 (37.2) | 81 (35.1) | 0.05 |
| **Functional ability** |  |  |  |  |  |  |  |
| Frailty (PRISMA-7) | 468 (59.8) | 210 (66.5) | 258 (55.4) | 0.23 | 148 (64.1) | 149 (64.5) | 0.01 |
| Fall in past year | 240 (30.7) | 112 (35.4) | 128 (27.5) | 0.17 | 84 (36.4) | 79 (34.2) | 0.06 |
| Multiple falls in past year | 109 (13.9) | 53 (16.8) | 56 (12) | 0.14 | 43 (18.6) | 39 (16.9) | 0.04 |
| Delirium (DSM-IV) | 244 (31.2) | 112 (35.4) | 132 (28.3) | 0.15 | 82 (35.5) | 76 (32.9) | 0.06 |
| **ICD-10 Co-Morbidities** |  |  |  |  |  |  |  |
| Cancer | 7 (0.9) | n<5 | ~ | 0.01 | n<5 | n<5 | 0.04 |
| Cerebrovascular disease | 85 (10.9) | 36 (11.4) | 49 (10.5) | 0.03 | 26 (11.3) | 28 (12.1) | 0.03 |
| Chronic kidney disease | 35 (4.5) | 19 (6.0) | 16 (3.4) | 0.12 | 10 (4.3) | 9 (3.9) | 0.02 |
| Chronic liver disease | n<5 |  |  | 0.06 | n<5 |  |  |
| Chronic lung disease | 108 (13.8) | 43 (13.6) | 65 (13.9) | 0.01 | 28 (12.1) | 35 (15.2) | 0.07 |
| Connective tissue disease | 30 (3.8) | 12 (3.8) | 18 (3.9) | 0.00 | 12 (5.2) | 7 (3.0) | 0.09 |
| Dementia | 61 (7.8) | 21 (6.6) | 40 (8.6) | 0.07 | 15 (6.5) | 20 (8.7) | 0.08 |
| Diabetes mellitus | 19 (2.4) | 10 (3.2) | 9 (1.9) | 0.08 | 8 (3.5) | n<5 |  |
| Heart failure | 94 (12.0) | 57 (18) | 37 (7.9) | 0.30 | 37 (16.0) | 32 (13.9) | 0.09 |
| Myocardial infarction | 90 (11.5) | 47 (14.9) | 43 (9.2) | 0.17 | 37 (16.0) | 32 (13.9) | 0.09 |
| Ulcer disease | 20 (2.6) | 5 (1.6) | 15 (3.2) | 0.11 | n<5 | 9 (3.9) |  |
| **Charlson Comorbidity Index** |  |  |  | 0.42 |  |  | 0.07 |
| 0 | 126 (16.1) | 29 (9.2) | 97 (20.8) |  | 28 (12.1) | 32 (13.9) |  |
| 1-2 | 332 (42.5) | 123 (38.9) | 209 (44.8) |  | 99 (42.6) | 103 (44.6) |  |
| ≥3 | 324 (41.4) | 164 (51.9) | 160 (34.3) |  | 115 (50.0) | 111 (48.1) |  |
| **Renal impairment (CrCl)** |  |  |  | 0.25 |  |  | <0.001 |
| Mild (50–80 mL/min) | 291 (37.2) | 98 (31) | 193 (41.4) |  | 75 (32.5) | 75 (32.5) |  |
| Moderate (30–49 mL/min) | 240 (30.7) | 112 (35.4) | 128 (27.5) |  | 84 (36.4) | 84 (36.4) |  |
| Severe (15–29 mL/min) | 109 (13.9) | 58 (18.4) | 51 (10.9) |  | 30 (13.0) | 30 (13.0) |  |
| ESRD (<15 mL/min) | 35 (4.5) | 13 (4.1) | 22 (4.7) |  | 9 (3.9) | 9 (3.9) |  |
| **Medication (ATC code)** |  |  |  |  |  |  |  |
| Anticoagulant (B01A) | 231 (29.5) | 151 (47.8) | 80 (17.2) | 0.69 | 102 (44.2) | 92 (39.8) | 0.11 |
| Antiplatelet (B01AC) | 448 (57.3) | 200 (63.3) | 248 (53.2) | 0.21 | 149 (64.5) | 142 (61.5) | 0.06 |
| Diuretic (C03) | 337 (43.1) | 183 (57.9) | 154 (33) | 0.52 | 95 (41.1) | 91 (39.4) | 0.03 |
| Antiarrhythmics, Class I and III (C01B) | 44 (5.6) | ~ | n<5 | 0.53 | ~ | n<5 |  |
| Beta blocking agents (C07A) | 394 (50.4) | 202 (63.9) | 192 (41.2) | 0.47 | 121 (52.4) | 117 (50.6) | 0.04 |
| Calcium channel blockers (C08) | 216 (27.6) | 92 (29.1) | 124 (26.6) | 0.06 | 63 (27.3) | 70 (30.3) | 0.07 |
| RAAS (C09) | 375 (48.0) | 158 (50) | 217 (46.6) | 0.07 | 115 (49.8) | 119 (51.5) | 0.04 |
| Lipid modifying agents, plain (C10A) | 535 (68.4) | 242 (76.6) | 293 (62.9) | 0.30 | 164 (71) | 162 (70.1) | 0.02 |
| NSAID (M01A) | 51 (6.5) | 15 (4.7) | 36 (7.7) | 0.12 | 14 (6.1) | 16 (7.0) | 0.02 |
| Antidepressants (N06A) | 212 (27.1) | 121 (38.3) | 91 (19.5) | 0.42 | 91 (39.4) | 68 (29.4) | 0.21 |
| Anti-dementia drugs (N06D) | 75 (9.6) | 31 (9.8) | 44 (9.4) | 0.01 | 22 (9.5) | 22 (9.5) | <0.001 |
| Hypnotics and sedatives (N05C) | 155 (19.8) | 71 (22.5) | 84 (18) | 0.11 | 52 (22.5) | 45 (19.5) | 0.07 |
| Analgesics (N02) | 311 (40.0) | 146 (46.2) | 165 (35.4) | 0.22 | 109 (47.2) | 114 (49.4) | 0.06 |
| **Polypharmacy** |  |  |  | 0.95 |  |  | <0.001 |
| *No (0–4 drugs)* | 61 (7.8) | n<5 | ~ |  | n<5 | n<5 |  |
| *Polypharmacy (5–9 drugs)* | 283 (36.2) | 58 (18.4) | 225 (48.3) |  | 58 (25.1) | 58 (25.1) |  |
| *Major polypharmacy (≥10 drugs)* | 438 (56.0) | 255 (80.7) | 183 (39.3) |  | 170 (73.6) | 170 (73.6) |  |
| **Narrow Therapeutic Index drug^†^** | 407 (52.0) | 201 (63.6) | 206 (44.2) | 0.40 | 127 (55.0) | 127 (55.0) | <0.001 |
| *Notes: Data reported as n (%), unless otherwise stated.*  *Abbreviations: ATC, anatomical therapeutic chemical; CrCl, creatinine clearance; DDI, drug-drug interaction; SMD, standardised mean difference.*  *†Carbamazepine, digoxin, flecainide, lithium, phenytoin, tacrolimus, theophylline, warfarin.*  ~Data further suppressed to prevent disclosure of data where n< 5. | | | | | | | |

## DDIs which Increase Bleeding Risk

### Study Population

For DDIs which increase the risk of bleeding, from the 782 ADAPT patients eligible for inclusion, 226 (n=113 DDI and n=113 non-DDI) were successfully matched. After propensity score matching, good overlap was achieved **(figure S4**), and confounders in the causal relationship between exposure to a DDI which increases bleeding risk and ADR-related hospital admission were well-balanced (**Table S2**). The mean age of this matched sample was 81.2 (±7.0) years; 49.6% were female; and 23.1% had polypharmacy, 76.1% with major polypharmacy; and 78.8% had at least one NTI drug (**Table S2**).

###


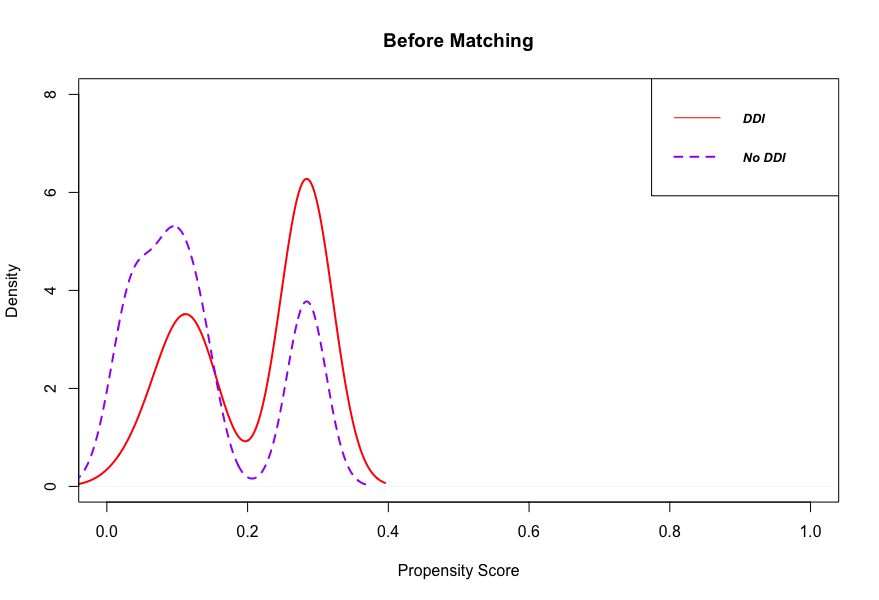

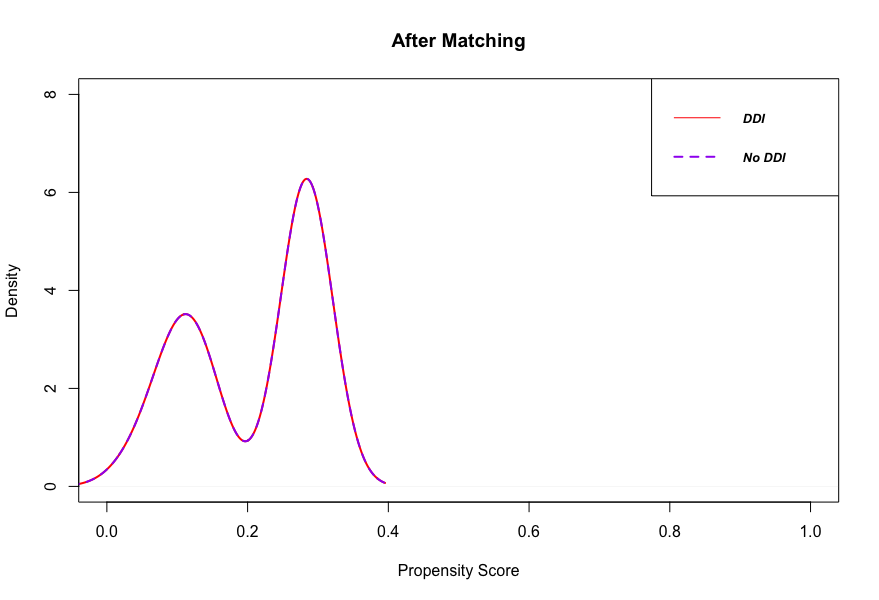


### **Figure S5.** Distribution of the estimated propensity score for DDI (with increased bleeding risk) exposure, before and after matching

### **Table S3.** Patient Characteristics Before and After Propensity Score Matching (DDI which Increases Bleeding Risk)

|  | **Before Matching** | | | | **After Matching** | | |
| --- | --- | --- | --- | --- | --- | --- | --- |
|  | **Overall (N=782)** | **DDI (n=113)** | **No DDI (n=669)** | **SMD** | **DDI (n=113)** | **No DDI (n=113)** | **SMD** |
| **Sociodemographics** |  |  |  |  |  |  |  |
| Age (mean [SD]) | 80.9 (±7.5) | 81.1 (±7.1) | 80.8 (±7.6) | 0.04 | 81.1 (±7.1) | 81.2 (±6.8) | 0.01 |
| *Female* | 408 (52.2) | 55 (48.7) | 353 (52.8) | 0.08 | 55 (48.7) | 57 (50.4) | 0.04 |
| Smoker | 456 (58.3) | 70 (61.9) | 386 (57.7) | 0.08 | 70 (61.9) | 71 (62.8) | 0.02 |
| Medical Card | 277 (35.4) | 46 (40.7) | 231 (34.5) | 0.13 | 46 (40.7) | 44 (38.9) | 0.04 |
| **Functional ability** |  |  |  |  |  |  |  |
| Frailty (PRISMA-7) | 468 (59.8) | 78 (69.0) | 390 (58.3) | 0.22 | 78 (69.0) | 75 (66.4) | 0.06 |
| Fall in past year | 240 (30.7) | 41 (36.3) | 199 (29.7) | 0.14 | 41 (36.3) | 39 (34.5) | 0.04 |
| Multiple falls in past year | 109 (13.9) | 16 (14.2) | 93 (13.9) | 0.01 | 16 (14.2) | 17 (15.0) | 0.03 |
| Delirium (DSM-IV) | 244 (31.2) | 38 (33.6) | 462 (69.1) | 0.06 | 38 (33.6) | 35 (31.0) | 0.08 |
| **ICD-10 Co-Morbidities** |  |  |  |  |  |  |  |
| Cancer | 7 (0.9) | 0 (0.0) | 7 (1.0) | 0.15 | 0 (0.0) | n<5 |  |
| Cerebrovascular disease | 85 (10.9) | 14 (12.4) | 71 (10.6) | 0.06 | 14 (12.4) | 14 (12.4) | <0.001 |
| Chronic kidney disease | 35 (4.5) | n<5 | ~ | 0.18 | n<5 | 13 (11.5) |  |
| Chronic liver disease | n<5 | ~ | n<5 |  | 0 (0.0) | n<5 |  |
| Chronic lung disease | 108 (13.8) | 14 (12.4) | 94 (14.1) | 0.05 | 14 (12.4) | 17 (15.0) | 0.08 |
| Connective tissue disease | 30 (3.8) | n<5 | ~ | 0.02 | n<5 | n<5 |  |
| Dementia | 61 (7.8) | 5 (4.4) | 56 (8.4) | 0.16 | 5 (4.4) | 6 (5.3) | 0.04 |
| Diabetes mellitus | 19 (2.4) | n<5 | ~ | 0.08 | n<5 | n<5 |  |
| Heart failure | 94 (12.0) | 24 (21.2) | 70 (10.5) | 0.30 | 24 (21.2) | 23 (20.4) | 0.03 |
| Myocardial infarction | 90 (11.5) | 28 (24.8) | 62 (9.3) | 0.42 | 28 (24.8) | 26 (23.0) | 0.06 |
| Ulcer disease | 20 (2.6) | n<5 | ~ | 0.06 | n<5 | n<5 |  |
| **Charlson Comorbidity Index** |  |  |  | 0.33 |  |  | 0.08 |
| 0 | 126 (16.1) | 11 (9.7) | 115 (17.2) |  | 11 (9.7) | 11 (9.7) |  |
| 1-2 | 332 (42.5) | 40 (35.4) | 292 (43.6) |  | 40 (35.4) | 43 (38.1) |  |
| ≥3 | 324 (41.4) | 62 (54.9) | 262 (39.2) |  | 62 (54.1) | 59 (52.2) |  |
| **Renal impairment (CrCl)** |  |  |  | 0.34 |  |  | 0.09 |
| Mild (50–80 mL/min) | 291 (37.2) | 32 (28.3) | 259 (38.7) |  | 32 (28.3) | 34 (30.1) |  |
| Moderate (30–49 mL/min) | 240 (30.7) | 39 (34.5) | 201 (30.0) |  | 39 (34.5) | 37 (32.7) |  |
| Severe (15–29 mL/min) | 109 (13.9) | 30 (26.5) | 79 (11.8) |  | 30 (26.5) | 28 (24.8) |  |
| ESRD (<15 mL/min) | 35 (4.5) | n<5 | ~ |  | n<5 | ~ |  |
| **Medication (ATC code)** |  |  |  |  |  |  |  |
| Anticoagulant (B01A) | 231 (29.5) | 105 (92.9) | 126 (18.8) | 2.24 | 105 (92.9) | 18 (15.9) | 2.426 |
| Antiplatelet (B01AC) | 448 (57.3) | 82 (72.6) | 366 (54.7) | 0.38 | 82 (72.6) | 82 (72.6) | <0.001 |
| Diuretic (C03) | 337 (43.1) | 66 (58.4) | 271 (40.5) | 0.36 | 66 (58.4) | 61 (54.0) | 0.09 |
| Antiarrhythmics, Class I and III (C01B) | 44 (5.6) | 22 (19.5) | 22 (3.3) | 0.53 | 22 (19.5) | n<5 |  |
| Beta blocking agents (C07A) | 394 (50.4) | 85 (75.2) | 309 (46.2) | 0.62 | 85 (75.2) | 81 (71.7) | 0.07 |
| Calcium channel blockers (C08) | 216 (27.6) | 32 (28.3) | 184 (27.5) | 0.02 | 32 (28.3) | 38 (33.6) | 0.10 |
| RAAS (C09) | 375 (48.0) | 62 (54.9) | 313 (46.8) | 0.16 | 62 (54.9) | 63 (55.8) | 0.02 |
| Lipid modifying agents, plain (C10A) | 535 (68.4) | 85 (75.2) | 450 (67.3) | 0.18 | 85 (75.2) | 92 (81.4) | 0.08 |
| NSAID (M01A) | 51 (6.5) | 7 (6.2) | 44 (6.6) | 0.02 | 7 (6.2) | n<5 |  |
| Antidepressants (N06A) | 212 (27.1) | 34 (30.1) | 178 (26.6) | 0.08 | 34 (30.1) | 30 (26.5) | 0.08 |
| Anti-dementia drugs (N06D) | 75 (9.6) | 8 (7.1) | 67 (10.0) | 0.11 | 8 (7.1) | 11 (9.7) | 0.09 |
| Hypnotics and sedatives (N05C) | 155 (19.8) | 22 (19.5) | 133 (19.9) | 0.01 | 22 (19.5) | 19 (16.8) | 0.07 |
| Analgesics (N02) | 311 (40.0) | 40 (35.4) | 271 (40.5) | 0.11 | 40 (35.4) | 42 (37.2) | 0.08 |
| **Polypharmacy** |  |  |  | 0.56 |  |  | <0.001 |
| *No (0–4 drugs)* | 61 (7.8) | n<5 | ~ |  | n<5 | n<5 |  |
| *Polypharmacy (5–9 drugs)* | 283 (36.2) | 26 (23.0) | 257 (38.4) |  | 26 (23.1) | 26 (23.1) |  |
| *Major polypharmacy (≥10 drugs)* | 438 (56.0) | 86 (76.1) | 352 (52.6) |  | 86 (76.1) | 86 (76.1) |  |
| **Narrow Therapeutic Index drug^†^** | 407 (52.0) | 89 (78.8) | 318 (47.5) | 0.68 | 89 (78.8) | 89 (78.8) | <0.001 |
| *Notes: Data reported as n (%), unless otherwise stated.*  *Abbreviations: ATC, anatomical therapeutic chemical; CrCl, creatinine clearance; DDI, drug-drug interaction; SMD, standardised mean difference. †Carbamazepine, digoxin, flecainide, lithium, phenytoin, tacrolimus, theophylline, warfarin.*  ~Data further suppressed to prevent disclosure of data where n< 5. | | | | | | | |

## Risk of ADR-Related Hospital Admission

In the propensity score matched groups, after adjusting for confounders, the risk of ADR-related hospital admission associated with any DDI exposure was estimated to be OR=1.18 [95% CI: 0.77-1.55]; and OR=2.31 [1.26, 3.78] for patients exposed to a DDI which increases the risk of bleeding.

## Cost of ADR-Related Hospital Admission

In the propensity score matched groups for patients with any severe potentially clinically important DDI, the median LOS in hospital was 7 [IQR 4-15] days, and the median cost of hospital admission was €6,160 [IQR 3520-13200]. Among patients with an ADR-related hospital admission, the difference in the cost of hospital admission was, on average, €746 [95% CI:-4,840, 6,332] higher for those with any severe DDI compared to those without a severe DDI. In the propensity score matched groups for patients with a DDI which increases the risk of bleeding, the median LOS in hospital was 7 [IQR 4-18] days, and the median cost of hospital admission was €6,160 [IQR 3520-15840]. Among patients with an ADR-related hospital admission, the difference in the cost of hospital admission was, on average, €3,030 [95% CI: -6,822, 12,881] higher for those with a DDI which increases the risk of bleeding compared to those without one of these DDIs (**Table S5**).

### **Table S5.** Sensitivity analysis using propensity score matching, cost (in Euro) of ADR-Related Hospital Admission by Type of DDI

|  | **Average Cost of ADR-Related Hospital Admission (€)** | **95% CI (€)** |
| --- | --- | --- |
| **Any DDI†** |  |  |
| Yes | 13,819 | [10,160, 18,795] |
| No | 13,073 | [9,907, 17,251] |
| Difference | 746 | [-4,840, 6,332] |
| **DDI which increases risk of bleeding** |  |  |
| Yes | 18,618 | [11,766, 29,460] |
| No | 15,589 | [11,381, 21,351] |
| Difference | 3,030 | [-6,822, 12,881] |
| *†Severe (i.e. the result may be a life-threatening event or have a permanent detrimental effect) potentially clinically important.* | | |
